# Recognition and processing of branched DNA substrates by Slx1–Slx4 nuclease

**DOI:** 10.1093/nar/gkz842

**Published:** 2019-10-04

**Authors:** Vineet Gaur, Weronika Ziajko, Shivlee Nirwal, Aleksandra Szlachcic, Marta Gapińska, Marcin Nowotny

**Affiliations:** Laboratory of Protein Structure, International Institute of Molecular and Cell Biology, 4 Trojdena St., 02-109 Warsaw, Poland

## Abstract

Structure-selective endonucleases cleave branched DNA substrates. Slx1 is unique among structure-selective nucleases because it can cleave all branched DNA structures at multiple sites near the branch point. The mechanism behind this broad range of activity is unknown. The present study structurally and biochemically investigated fungal Slx1 to define a new protein interface that binds the non-cleaved arm of branched DNAs. The DNA arm bound at this new site was positioned at a sharp angle relative to the arm that was modeled to interact with the active site, implying that Slx1 uses DNA bending to localize the branch point as a flexible discontinuity in DNA. DNA binding at the new interface promoted a disorder-order transition in a region of the protein that was located in the vicinity of the active site, potentially participating in its formation. This appears to be a safety mechanism that ensures that DNA cleavage occurs only when the new interface is occupied by the non-cleaved DNA arm. Models of Slx1 that interacted with various branched DNA substrates were prepared. These models explain the way in which Slx1 cuts DNA toward the 3′ end away from the branch point and elucidate the unique ability of Slx1 to cleave various DNA structures.

## INTRODUCTION

DNA repair is an important biological process that ensures genome stability. Cells rely on an intricate network of DNA repair pathways to counteract various types of DNA damage. One of the most deleterious types of damage is the DNA double-strand break. Double-strand breaks can form when two single-strand breaks are in close proximity, after the attempted replication of nicked DNA or upon replication fork collapse. If double-strand breaks are not repaired, then this can lead to the loss of entire segments of genetic information. Homologous recombination is generally considered an error-free pathway of double-strand break repair because it utilizes a homologous sequence as the template for DNA repair. Homologous recombination invariably involves the generation of joint (branched) DNA intermediates (e.g. replication forks [RFs], splayed-arms, 5′-flaps, 3′-flaps and Holliday junctions [HJs]) (1). The structures of these joint DNA molecules vary significantly but must be converted into linear DNA duplexes to ensure accurate homologous recombination and genome stability. One of the mechanisms of joint DNA molecule processing employs structure-selective endonucleases (SSEs) (1–3).

Structure-selective endonucleases belong to various nuclease families, including the FEN1/XPG, XPF/ERCC4 and GIY-YIG families. As the name implies, these enzymes cleave specific types of branched DNA structures, as opposed to specific DNA sequences. Most of these enzymes have defined substrate specificities and thus play roles in distinct pathways of DNA repair (1,4,5). For example, XPF-ERCC1 participates in interstrand cross-link repair and nucleotide excision repair (6,7) where it cleaves on the 5′-side of fork structures that contain interstrand crosslinks and nucleotide excision repair DNA bubbles, respectively (8–12). MUS81-EME1 belongs to the same nuclease family as XPF-ERCC1, adopts a similar structure, and plays an important role in homologous recombination. The preferred DNA substrates of MUS81-EME1 are RFs and nicked HJs (13,14). Substrate specificity is largely determined by a structural element, termed the 5′-phosphate-binding pocket, and a hydrophobic wedge that positions the DNA substrates for cleavage. The presence of a 5′-phosphate pocket confers specificity to bind a 3′-flap substrate over a 5′-flap substrate (15). GEN1 is a Holliday junction resolvase which belongs to FEN/XPG family. It interacts with all four arms of the Holliday junctions for specific binding and a chromodomain mediates additional DNA interaction (16,17). SLX1 belongs to the GIY-YIG superfamily of nucleases. This superfamily comprises type II restriction endonucleases, homing nucleases, and UvrC endonuclease of the prokaryotic nucleotide excision repair pathway (18). SLX1 is unique among SSEs because it is a very promiscuous endonuclease. When bound to SLX4, SLX1 cleaves various branched DNA substrates near the branch point (19). In contrast to XPF-ERCC1 and MUS81-EME1, which specifically nick duplex DNA on the 5′-side of a single-stranded/double-stranded DNA branch point, SLX1–SLX4 can incise duplex or single-stranded DNA on either the 5′- or 3′-sides of the branch point (14,20). For example, within the context of a model RF, SLX1–SLX4 can nick either the leading or lagging strand template (14). However, the mechanism that underlies this promiscuous endonuclease activity is unknown.

Interestingly, some SSEs function in a coordinated manner to utilize their distinct substrate specificities. One prominent example is cooperation between XPF-ERCC1, MUS81-EME1 and SLX1, which are brought together via their interactions with the SLX4 platform protein (14,20). Nucleases that associate with SLX4 have been shown to cooperate in HJ resolution. SLX1 introduces the first nick, and MUS81-EME1 rapidly cleaves the nicked HJ (20). XPF-ERCC1 enhances this activity through a poorly understood but non-catalytic mechanism (14).

We previously reported the mechanism by which the Slx4 scaffold activates Slx1 nuclease. In the absence of Slx4, Slx1 adopts a homodimeric architecture that occludes the active site. Conversely, Slx4 disrupts the inhibitory Slx1 homodimer and forms a catalytically active Slx1–Slx4 heterodimer. Guided by structural similarities between the active site of Slx1 and GIY-YIG type II restriction endonucleases, we proposed a possible mode of DNA binding by Slx1 (21). In contrast to other SSEs, very few structural elements can be predicted that may facilitate the binding of branched DNA substrates by Slx1. In fact, our previous studies were only able to identify scattered patches of surface-exposed basic residues as important elements of DNA binding (21). As such, the structural and mechanistic basis of the broad substrate specificity of Slx1 remained unknown.

The present study investigated the mechanism of DNA binding by Slx1 and defined a new DNA-binding interface. The positioning of this interface explains the way in which the enzyme utilizes helical discontinuity in branched DNA substrates as the main determinant for incision point selection. We propose a mechanism that explains the ability of Slx1–Slx4 to cleave various DNA joint molecules that are intermediates during DNA repair.

## MATERIALS AND METHODS

### Protein expression and purification

Synthetic genes for *Candida glabarata* Slx1 (*Cg*-Slx1) and the conserved C-terminal domain of Slx4 (*Cg*-Slx4^CCD^) (residues 557–726) were codon-optimized for expression in *Escherichia coli* and obtained from Bio Basic Inc., Canada. Synthetic genes for *Thielavia terrestris* Slx1 (*Tt*-Slx1) and a fragment of Slx4^CCD^ (*Tt*-Slx4^CCD3^) (residues 834–936) were codon-optimized for expression in *E. coli* and obtained from GenScript^®^. *Cg*-Slx1 and *Cg-*Slx4^CCD^ were subcloned into a pET28a vector (Novagen) with an N-terminal 6xHis-SUMO tag. *Tt*-Slx1 and *Tt*-Slx4^CCD3^ were subcloned into a pRSFduet-1 vector (Novagen) using multiple cloning site I with an N-terminal 6XHis tag and multiple cloning site II without a tag, respectively. All point substitutions were introduced by QuikChange Site-Directed Mutagenesis (Agilent Technologies). All proteins (wild type and mutants) were expressed in *E. coli* BL21 (DE3) Rosetta (Novagen). For protein expression, bacteria were grown in LB medium at 37°C, induced with 0.4 mM isopropyl β-d-1-thiogalactopyranoside at OD_600_ = 0.7–0.9, and grown overnight at 18°C. The cells were harvested by centrifugation, and pellets were washed with phosphate-buffered saline (PBS) prior to purification.

A complex of *Cg*-Slx1 and *Cg*-Slx4^CCD^ (wild type and point substitution variants) was purified as described earlier (21). A complex of *Tt*-Slx1 and *Tt*-Slx4^CCD3^ was purified by resuspending the pellet that contained proteins that were co-expressed from the pRSFduet-1 vector in lysis buffer (20 mM Tris–HCl [pH 8.5], 500 mM NaCl, 5 mM imidazole, 10% [v/v] glycerol, and 5 mM 2-mercaptoethanol) and lysed by sonication. The lysate was centrifuged at 186 000 × *g* at 4°C for 40 min, and the supernatant was loaded onto a His-Trap HP column (GE Healthcare) that was equilibrated with lysis buffer. Protein was eluted using a linear gradient of imidazole from 5 to 500 mM. The fractions were analyzed by sodium dodecyl sulfate-polyacrylamide gel electrophoresis. Fractions that contained a complex of *Tt*-Slx1 and *Tt*-Slx4^CCD3^ were further purified using a Superdex-200 size exclusion column (GE Healthcare) in a buffer that contained 20 mM Tris–HCl (pH 8.5), 10% (v/v) glycerol, and 5 mM 2-mercaptoethanol. Fractions that contained the *Tt*-Slx1–Slx4^CCD3^ complex were pooled and concentrated using a 30k MWCO Amicon Ultra Centrifugal Filter Device (Millipore).

### Crystallization

The active-site mutant of *Tt*-Slx1^E79Q^-Slx4^CCD3^ at a concentration of 13 mg/ml was crystallized in the presence of either HJs or self-annealing splayed-arm substrate in a buffer that contained 20 mM Tris–HCl (pH 8.5), 100 mM NaCl and 5 mM ethylenediaminetetraacetic acid. The oligonucleotide sequences are presented in Supplementary Table S1. DNA substrate compositions are presented in Supplementary Table S2. For crystallization, the protein-to-DNA molar ratio was 1:0.5. Both crystal forms were obtained in a buffer that contained 0.1 M MES (pH 6.0), 0.2 M calcium chloride dihydrate and 20% (v/v) PEG 6000 using the sitting-drop vapor diffusion method at room temperature. The conditions were obtained by screening using PACT premier HT-96 (Molecular Dimensions). Crystals of both types were cryoprotected with 30% (v/v) glycerol before data collection.

### Diffraction data collection, structure solution and refinement

Diffraction data for crystals that were grown in the presence of HJs (dataset 1) were collected at Diamond Light Source at a wavelength of 1.2828 Å. These crystals diffracted to a maximum resolution of 3.2 Å. Diffraction data for crystals with splayed-arm substrate (dataset 2) were collected at beamline 14.1 at Berliner Elektronenspeicherring-Gesellschaft für Synchrotronstrahlung (BESSY) (22) at a wavelength of 0.9184 Å. These crystals diffracted to a maximum resolution of 2.7 Å. Diffraction data were processed and scaled using XDS (23). Phases for dataset 1 were determined using single-wavelength anomalous diffraction for Zn in the AutoSol module of Phenix (24). Phases for dataset 2 were determined using molecular replacement in the Phaser-MR module of Phenix (25). The refined structure of *Tt*-Slx1^E79Q^-Slx4^CCD3^ was used as the starting model for molecular replacement for dataset 2. Coot was used for iterative model building (26,27). The models were refined using Phenix, with *R*_free_, calculated with 5% unique reflections. Models from dataset 1 and dataset 2 were refined with 95.4% and 97.7% residues in the favored region of the Ramachandran plot, respectively. Structure validation was performed using MolProbity (28). The structural analysis was performed using PyMol (version 3.3.0, Schrodinger). The buried surface area was calculated using PDBe PISA (29). The diffraction statistics are summarized in Table 1. The structures of *Tt*-Slx1^E79Q^-Slx4^CCD3^ alone and in complex with the DNA were deposited in the PDB under the accession codes 6SEH and 6SEI, respectively.

**Table 1.** Data collection and refinement statistics

Data collection	*Tt*-Slx1–Slx4^CCD3^	*Tt*-Slx1–Slx4^CCD3^-DNA
Space group	*C* 2	*P* 2_1_
Cell dimensions		
*a*, *b*, *c* (Å)	180.3 63.4 103.7	60.1 92.8 93.3
α, β, γ (°)	90 116.7 90	90 91.6 90
Resolution (Å)	29.74–3.15 (3.34–3.15)	46.61–2.69 (2.79–2.69)
CC_1/2_	99.4 (77.2)	98.5 (82.0)
Mean *I* / σ*I*	8.75 (1.72)	7.23 (2.44)
Completeness (%)	97.0 (90.0)	98.9 (98.9)
Redundancy	3.41 (3.38)	3.49 (3.59)
**Refinement**		
Resolution (Å)	29.74–3.15	46.61–2.69
No. of unique reflections	17 420	28 286
*R*_work_/*R*_free_ (%)	20.2/25.4	20.8/26.6
No. atoms	5067	6536
Protein	5053	5607
Nucleic acid		647
Ion/Water	14	282
*B*-factors (Å^2^)	99.3	38.6
Protein	99.3	33.9
Nucleic acid		83.6
Ion/water	79.5	28.1
RMSD		
Bond length (Å)	0.003	0.004
Bond angle (°)	0.577	0.637

Statistics for the highest-resolution shell are shown in parentheses.

### Nuclease assay

Synthetic DNA substrates were prepared by annealing appropriate DNA oligonucleotides that were synthesized by Metabion. The sequences of the oligonucleotides are presented in Supplementary Table S1, and the oligonucleotides that were used to generate specific DNA substrates are presented in Supplementary Table S2. Nuclease assays were performed with a protein concentration of 125 nM and a DNA substrate concentration of 250 nM in a reaction that contained 50 mM Tris–HCl (pH 8.5), 100 mM NaCl, 0.1 mg/ml bovine serum albumin, 4 mM MgCl_2_, 1 mM DTT and 0.5% glycerol. The substrates comprised 200 nM unlabeled DNA substrate and 50 nM DNA that was labeled with fluorescein on X0-1 and Cy5 on X0-4. The samples were then taken out at various time points and stopped by adding equal amounts of 100% formamide that contained Orange G dye and boiling the samples for 5 min at 95°C. The samples were run on a 20% TBE-Urea polyacrylamide gel at a constant power of 18 W for 40 min. The cleavage sites were mapped using fluorescein- or Cy5-labeled DNA marker oligonucleotides of various lengths (Supplementary Table S2).

### Fluorescence anisotropy

The binding of 5′-flap DNA substrate to *Cg*-Slx1–Slx4^CCD^ and its variants was studied using fluorescence anisotropy. The 5′ -flap substrate was labeled with fluorescein at the 5′-end of X0-1 and Cy5 at the 3′-end of X0-4. Substrates were used at a concentration of 25 nM, and the protein concentration ranged from 0 to 500 nM. Binding was studied in a buffer that contained 20 mM HEPES (pH 7.5), 2.5 mM CaCl_2_, 100 mM NaCl and 0.1 mg/ml bovine serum albumin at 25°C. Binding reactions were set in Corning 96 flat bottom black polystyrene plates. Anisotropy was measured using a Tecan Infinite M1000 microplate reader at an excitation wavelength of 635 nm and emission wavelength of 670 nm.

### Fourier-transform infrared spectroscopy

Secondary structure analysis was performed using a Bruker Tensor 27 spectrometer. Protein samples were used at a concentration of 0.5–1.0 mg/ml in 20 mM HEPES (pH 7.5) and 150 mM NaCl. The results were analyzed using OPUS-PRO software.

## RESULTS

### Overall structure

Our aim was to understand the mechanism that underlies the broad specificity of Slx1 for various branched DNA substrates. To achieve this, we sought to obtain high-resolution structural information for Slx1 in complex with different DNA substrates. Multiple crystallization trials were designed for complexes of Slx1 with a conserved C-terminal domain of Slx4 (Slx4^CCD^) from several fungal species in the presence of various DNA substrates (i.e. HJs, 5′ flaps, 3′ flaps, splayed arms and nicked DNA substrates). We obtained two crystal forms of Slx1–Slx4^CCD3^ from *Thielavia terrestris* (*Tt*-Slx1–Slx4^CCD3^, where CCD3 corresponds to a previously characterized *Candida glabrata* construct) (21). One form was grown in the presence of HJs, and the other was grown with splayed-arm DNA. Crystals of *Tt*-Slx1–Slx4^CCD3^ that were obtained in the presence of HJs were found to be crystals of protein alone. These *apo* crystals crystallized in the *C* 2 space group and diffracted X-rays to a resolution of 3.1 Å (Table 1). The phases were determined using single-wavelength anomalous diffraction (SAD) for zinc ions at a wavelength of 1.2828 Å. The asymmetric unit contained two molecules. Slx1 comprises two distinct domains: N-terminal GIY-YIG nuclease domain and C-terminal RING domain which binds two zinc ions. The CCD3 fragment of Slx4 bound between these two domains (Supplementary Figure S1A). The overall structure of *Tt*-Slx1–Slx4^CCD3^ was very similar to Slx1–Slx4^CCD3^ from *Candida glabrata*, which was reported earlier (21). The structures of *Cg* and *Tt-*Slx1 could be superimposed with a root-mean-square deviation (rmsd) of 2.3 Å over 142 C-α atoms (Supplementary Information, Figure S1B). The main difference between them was the connection between the GIY-YIG and RING domains. This region formed a long α-helix in *Cg-*Slx1–Slx4^CCD3^, whereas it lacked a secondary structure and was partly disordered in *Tt*-Slx1–Slx4^CCD3^ (Supplementary Figure S1). The structures of *Tt*- and *Cg*-Slx4^CCD3^ were nearly identical, with an rmsd of 1.1 Å over 45 C-α atoms.

Crystals of *Tt*-Slx1–Slx4^CCD3^ that were grown in the presence of splayed-arm substrate belonged to the *P*2_1_ space group. These crystals diffracted to a resolution of 2.7 Å. Phases were determined by molecular replacement using the *apo* structure as the search model. Clear electron densities were observed for DNA that was bound by the protein (Supplementary Figure S2A). The overall conformation of *Tt*-Slx1–Slx4^CCD3^ in complex with splayed-arm substrate was the same as the *apo* structure without any significant interdomain movements to accommodate the substrate. Unexpectedly, the DNA substrate, which was designed to adopt a splayed-arm conformation, was found to form a double-helical conformation, in which the two single-stranded overhangs were base-paired because of partial complementarity, forming a slightly distorted double helix with a 2-nt bulge and a G-A mismatch (Supplementary Figure S3).

### Structures reveal a new DNA-binding interface

**Figure 1.**
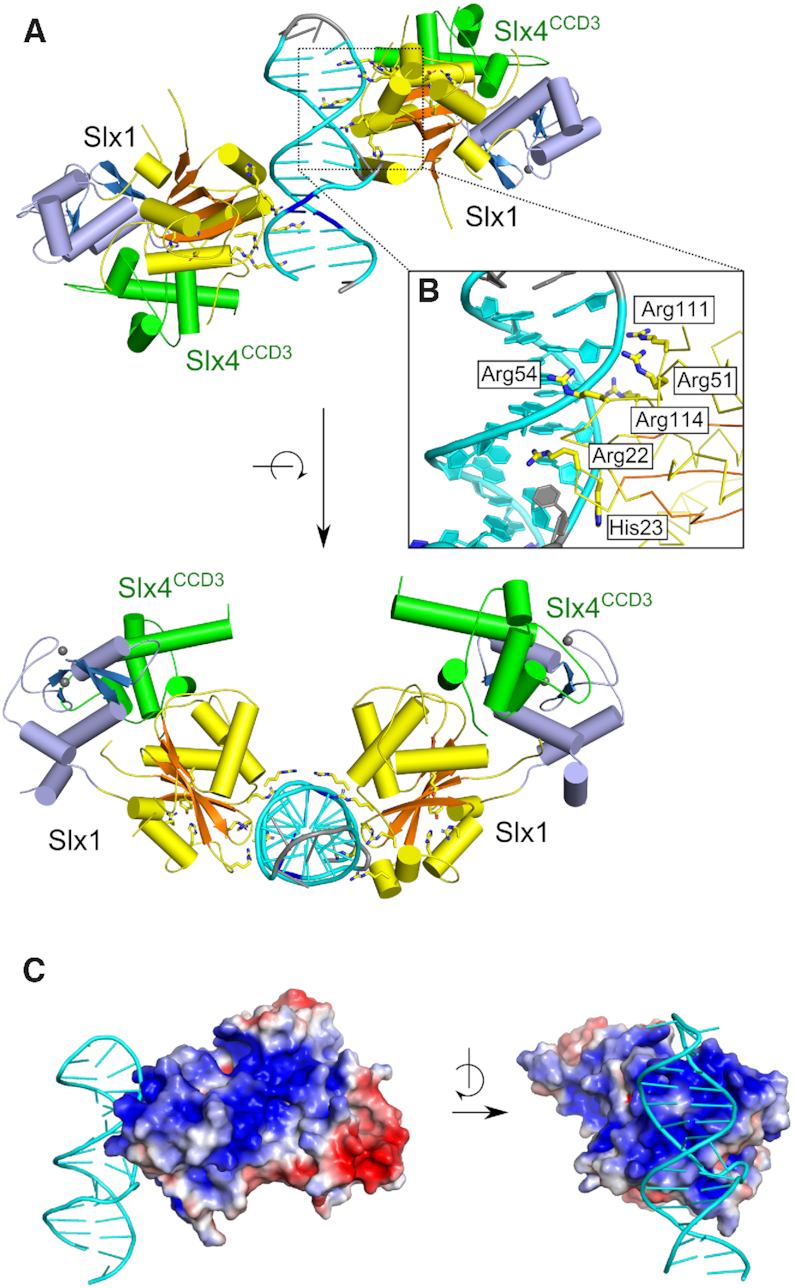
Structure of *Tt*-Slx1–Slx4^CCD3^ in complex with DNA. (**A**) Overall structure (two views). Slx1 is shown in yellow/orange for GIY-YIG domain and blue for RING domain. Slx4^CCD3^ is shown in green. DNA is shown in cyan for canonical base pairs, in blue for single G-A mismatch and in gray for unpaired bases (see Supplementary Figure S3 for details). Residues that are involved in DNA-binding are shown as sticks. (**B**) Close-up view of the DNA interface. (**C**) Surface charge distribution. The protein surface is colored from red (negative) to blue (positive) charge (±3 kT/e).

The asymmetric unit of *Tt*-Slx1–Slx4^CCD3^-DNA crystals contained one DNA molecule that was bound by two protein molecules (Figure 1A). The DNA-binding interface in each protein was identical and located on the side of the GIY-YIG domain at the edge of the central β-sheet. The protein bound the DNA backbone through basic amino acid side chains (R22, H23, R51, R54, K101 and R102; Figure 1B) with a buried surface area of 637.3 Å^2^ in the better-organized protein–DNA interface. These DNA-binding residues are conserved among fungal Slx1 proteins (Supplementary Figure S4). As expected, the DNA-binding interface was overall positively charged (Figure 1C).

We previously used structural similarities between Slx1 and GIY-YIG type II restriction endonucleases to predict the interface for catalytic DNA binding by *Cg*-Slx1 (21). The predicted interface comprised two sites: site I (R35, R38 in *Cg*-Slx1) and site II (H80, H84 in *Cg*-Slx1). In the new *Tt*-Slx1–Slx4^CCD3^-DNA structure, the DNA was located away from the active site and occupied a markedly different position from the position that we predicted earlier. Thus, our *Tt*-Slx1–Slx4^CCD3^-DNA structure revealed an additional and novel DNA-binding interface. We designated this newly identified DNA-binding surface as site III.

**Figure 2.**
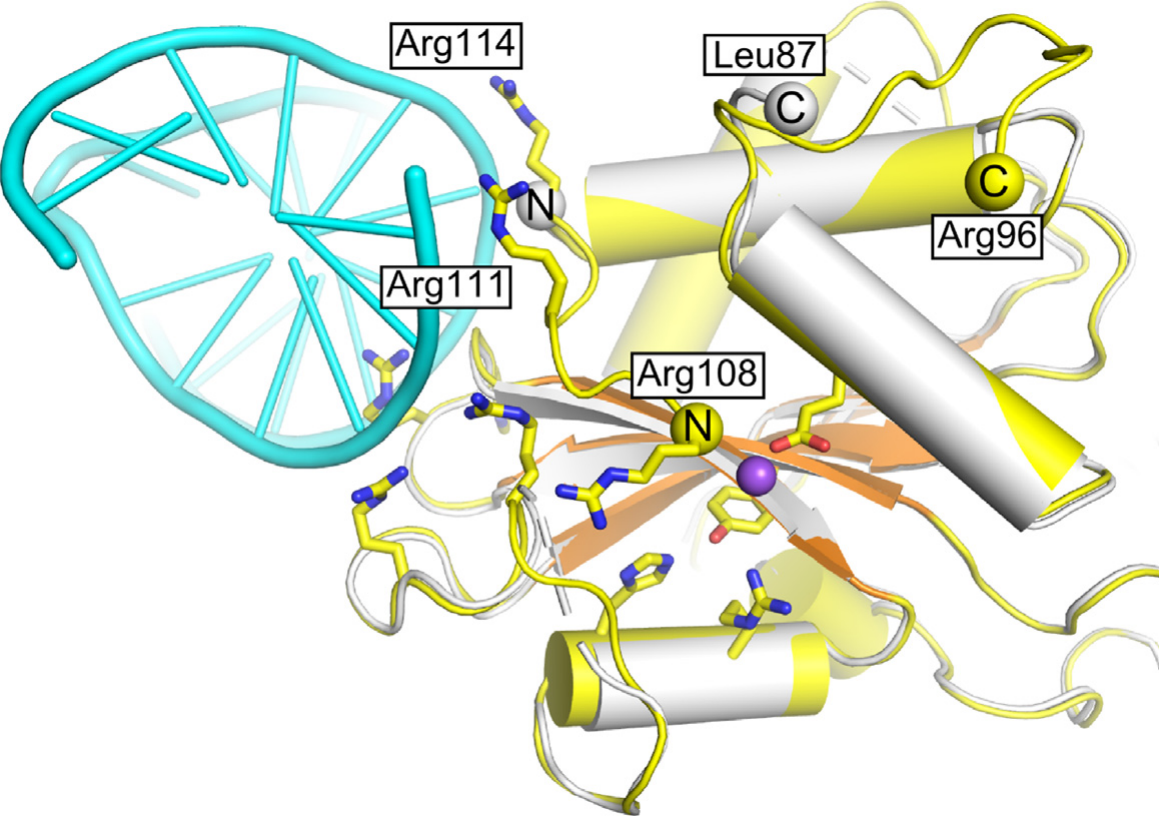
Structural differences at the new DNA-binding interface between *apo* and DNA-bound *Tt*-Slx1. The DNA-bound structure is shown in yellow/orange for protein and cyan for DNA. DNA binding residues, and active site residues are shown as yellow sticks. The superimposed *apo* structure is shown in white. Yellow/white spheres indicate termini that flank regions that are not visible in the structures. The position of the catalytic metal ion, modeled based on the structure of Hpy188I-DNA complex (PDB ID: 3OQG) (39), is shown as a purple sphere.

Comparisons of the *apo* and *Tt*-Slx1–Slx4^CCD3^-DNA structures revealed intriguing structural differences. Two residues that formed important DNA contacts, R111 and R114, were located in a loop that could be visualized in the protein-DNA complex (Figure 2, Supplementary Figure S2B). In the *apo* structure, the region that comprised residues 88–113 was not visible in the electron density maps and was likely disordered. In the DNA-bound structure, the N-terminal flank of this gap could be traced to R108. Therefore, upon DNA binding at site III, a part of this region that comprised residues 108–113 became ordered. R108 was located very close to the active site of the enzyme and potentially within coordination distance of the predicted position of catalytic metal ion. We hypothesize that the ordering of this region is coupled to the proper assembly of the active site, which could serve as a safety latch mechanism (i.e. catalysis could only occur when DNA is bound at site III). Further studies will be necessary to confirm this potential mechanism. We also note that the region that comprised residues 97–107 was still disordered in the DNA-bound structure. This region may become ordered when DNA binds to site I around the active site of the nuclease, potentially forming another safety mechanism.

### Model of the complex with branched DNA reveals DNA bending as the key determinant of specificity

**Figure 3.**
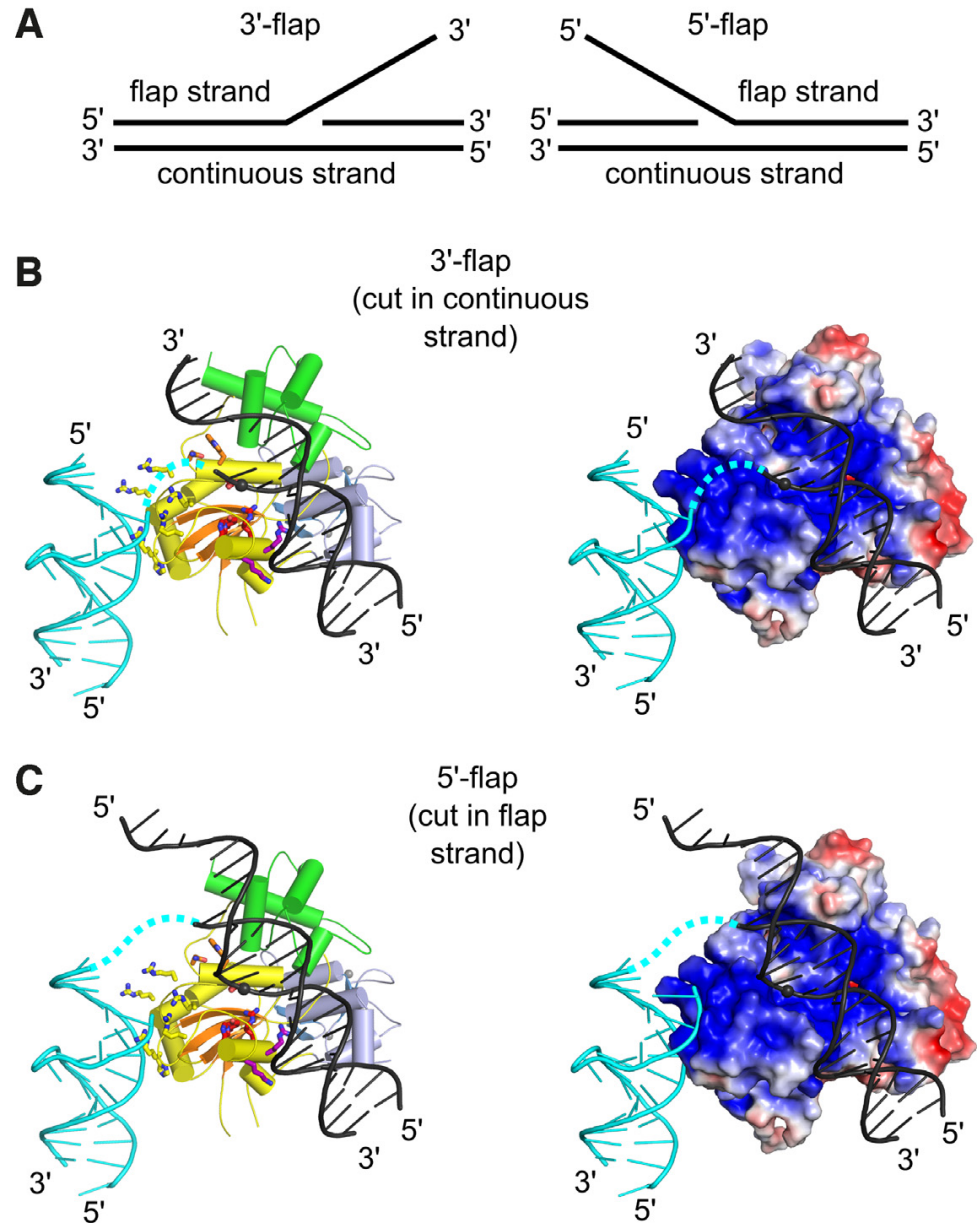
Models of Slx1–Slx4^CCD3^ interactions with branched DNA. (**A**) Schematic of the DNA flap substrates with terminology of the strands. (**B**) Model of Slx1–Slx4^CCD3^ bound to a 3′-flap substrate in configuration, which is conducive to incision in the continuous strand. (Left) Slx1 GIY-YIG domain is shown in yellow with β-strands in orange and RING domain is shown in blue. Slx4^CCD3^ is shown in green. The modeled DNA is based on the structure of R.Eco29kI restrictase (PDB ID: 3NIC) (40) and is shown in black with the scissile phosphate shown as a sphere. A fragment of the DNA that is observed in the *Tt*-Slx1–Slx4^CCD3^-DNA structure is shown in cyan. The potential link between the two DNA double helices is shown as a dashed cyan line. Residues of the active sites are shown as red sticks. Residues of site I, II and III are shown as purple, orange and yellow sticks, respectively. (Right) The same model with protein in surface representation, colored according to the surface potential (±3 Kt/e). (**C**) Model of Slx1–Slx4^CCD3^ bound to a 5′-flap substrate, in configuration which is conducive to incision of the flap strand. The representations are the same as in (B).

We next analyzed the way in which the new DNA-binding interface (i.e. site III) is related to the previously postulated interface around the Slx1 active site. We superimposed the catalytic structures of GIY-YIG type II restriction endonuclease on our new structure of DNA-bound *Tt*-Slx1–Slx4^CCD3^. The DNA molecules from GIY-YIG complexes were used to model nucleic acid binding at and around the active center and site I. This analysis revealed that the two interfaces did not overlap, and the two DNA duplexes were at an angle of ∼50° (Figure 3). Interestingly, this analysis could also be used to readily prepare models of complexes of Slx1 with various branched substrates. This could be achieved when pairs of 5′ and 3′ termini were linked with single-stranded DNA (ssDNA) in the region where the two double-stranded helices (which were observed in the structure and modeled) meet (Figure 3). For example, when the 5′ -end of the DNA strand that was located at the active site was linked to the 3′-end of the DNA in the new interface (site III), the linking DNA could be neatly accommodated in a positively charged channel on the protein surface. The resulting DNA geometry corresponded to the cut of the 3′-flap in the continuous (non-flap) strand (Figure 3A and B). Similarly, the 3′ end of the DNA at site I could be connected by a longer DNA loop to the 5′-end of the DNA at site III, resulting in a model of Slx1 that cleaved the 5′-flap (Figure 3C).

Our models explain the way in which Slx1 is able to accommodate various substrates and cleave them in multiple positions. The key feature of Slx1 is that it introduces cuts near the branch point. Our models suggest that this is achieved by bending the DNA substrate, such that Slx1 recognizes the branch point as a flexible discontinuity in the DNA. By interacting with two arms of the DNA substrate, Slx1 is able to bind and cleave various branched substrates.

### Mapping of DNA cuts confirms the structural models

**Figure 4.**
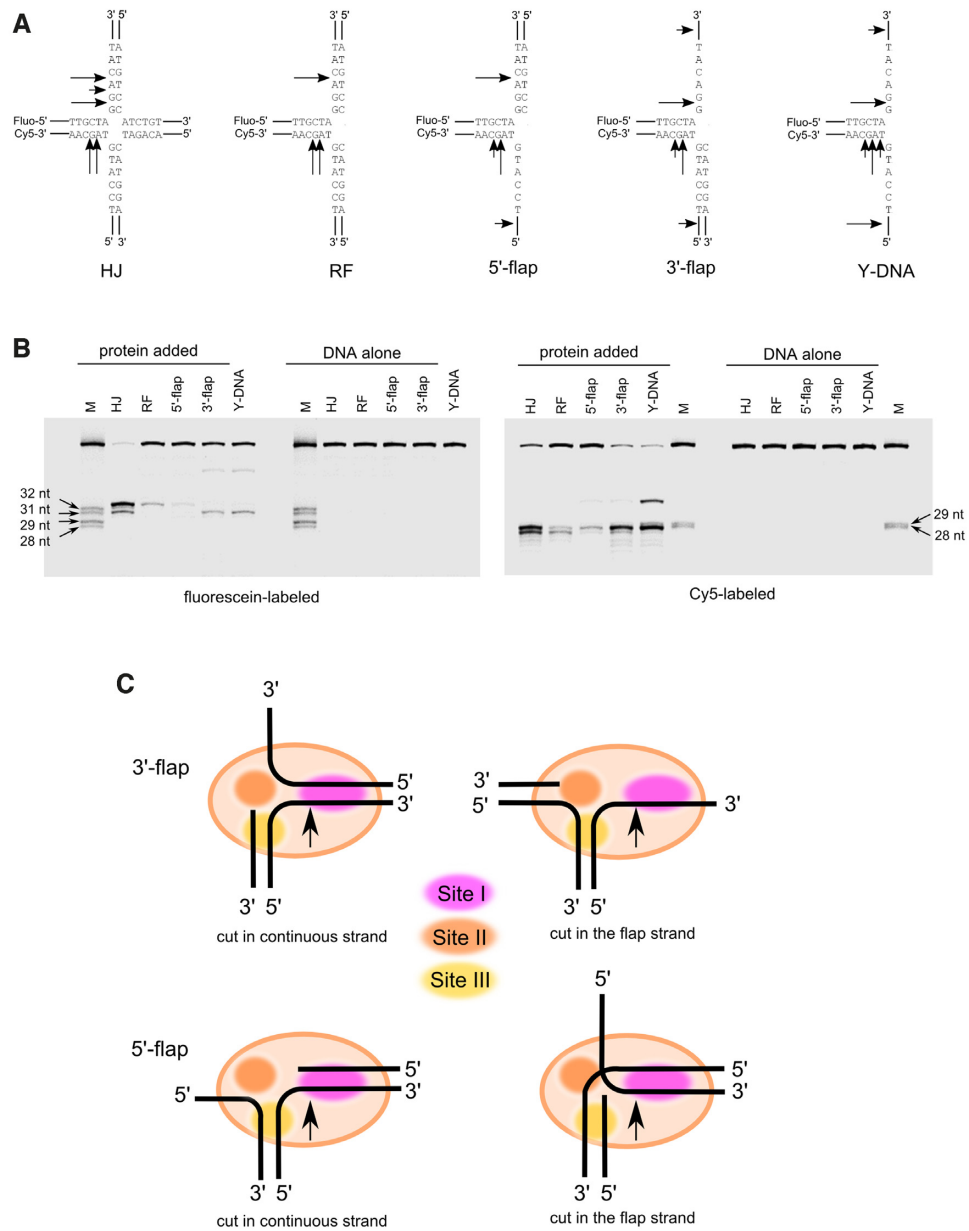
Mapping of DNA cuts by Slx1–Slx4^CCD^ in various branched DNA substrates. (**A**) Schematics of the substrates that were used, with arrows indicating the observed cut sites. (**B**) Fluorescently labeled substrates that are indicated above each lane were run alone or after mixing with *Cg*-Slx1–Slx4^CCD^_._ Cleavage products were analyzed by denaturing polyacrylamide gel electrophoresis. (Left) Fluorescein signal. (Right) Cy5 signal. M, size markers, the size of which is indicated next to the gel. (**C**) Schematic of the various modes of the interaction between branched substrates and Slx1–Slx4. The positions of sites I-III are indicated with colored ovals.

We next sought to verify these models by mapping the sites of the DNA cuts that were introduced by Slx1–Slx4^CCD^. These experiments were performed using *C. glabrata* protein, for which substantial structural and biochemical information is available (21). The following substrates were used: HJ, RF, 5′-flap, 3′-flap and splayed-arm DNA (Y-DNA) (Figure 4A). In these substrates, two oligonucleotides were labeled, one at the 5′-end with fluorescein and the other at the 3′-end with Cy5. After incubation with *Cg*-Slx1–Slx4^CCD^, the cleavage products were resolved under denaturing conditions to map the cut sites. This analysis revealed that *Cg*-Slx1–Slx4^CCD^ cut the HJ substrate both in fluorescein- and Cy5-labeled strands. The cuts were located 1–3 nt from the branch point toward the 3′-end of the cleaved strand (Figure 4A and B). Similar patterns were observed for the RF and 5′-flap substrates, with a predominant cut 3 nt from the branch point on the fluorescein-labeled strand and 2 or 3 nt from the branch point on the Cy5-labeled strand. Similar to HJs, the cut was located on the 3′-side of the branch point (Figure 4A and B). For 3′-flap and Y-DNA substrates, the pattern was similar, but the cut in the fluorescein-labeled strand was 1 nt from the branch point (Figure 4A and B). Minor products reflected less favorable cut sites that were located further downstream of the branch point.

The observed patterns agreed well with the models of Slx1–Slx4 that was bound to branched DNA substrates (Figure 4), particularly the fact that the cuts were located on the 3′ side of the branch point. The model predicted that in the duplex that was bound at site I, the strand that interacted with the active site ran in a 5′ to 3′ direction away from the area where the modeled DNA and DNA from our new crystal structures met. Thus, the cut site should be located away from the branch point toward the 3′ end of the cleaved strand. Notably, the cuts in ssDNA (in 3′-flap or in the 3′-arm of splayed-arm DNA) occurred closer to the branch point. We assumed that this would result from placing ssDNA at site I. Such ssDNA could reach the active site in extended and not helical form, and phosphates that were closer to the branch point would interact with the active center for cleavage (Figure 4C). The cut sites that were observed in our experiments agreed very well with the cut sites that were obtained for human protein (14,20), suggesting that the mechanism that is described herein for fungal protein also operates for its human counterpart.

### Mutagenesis studies confirm the structural models

**Figure 5.**
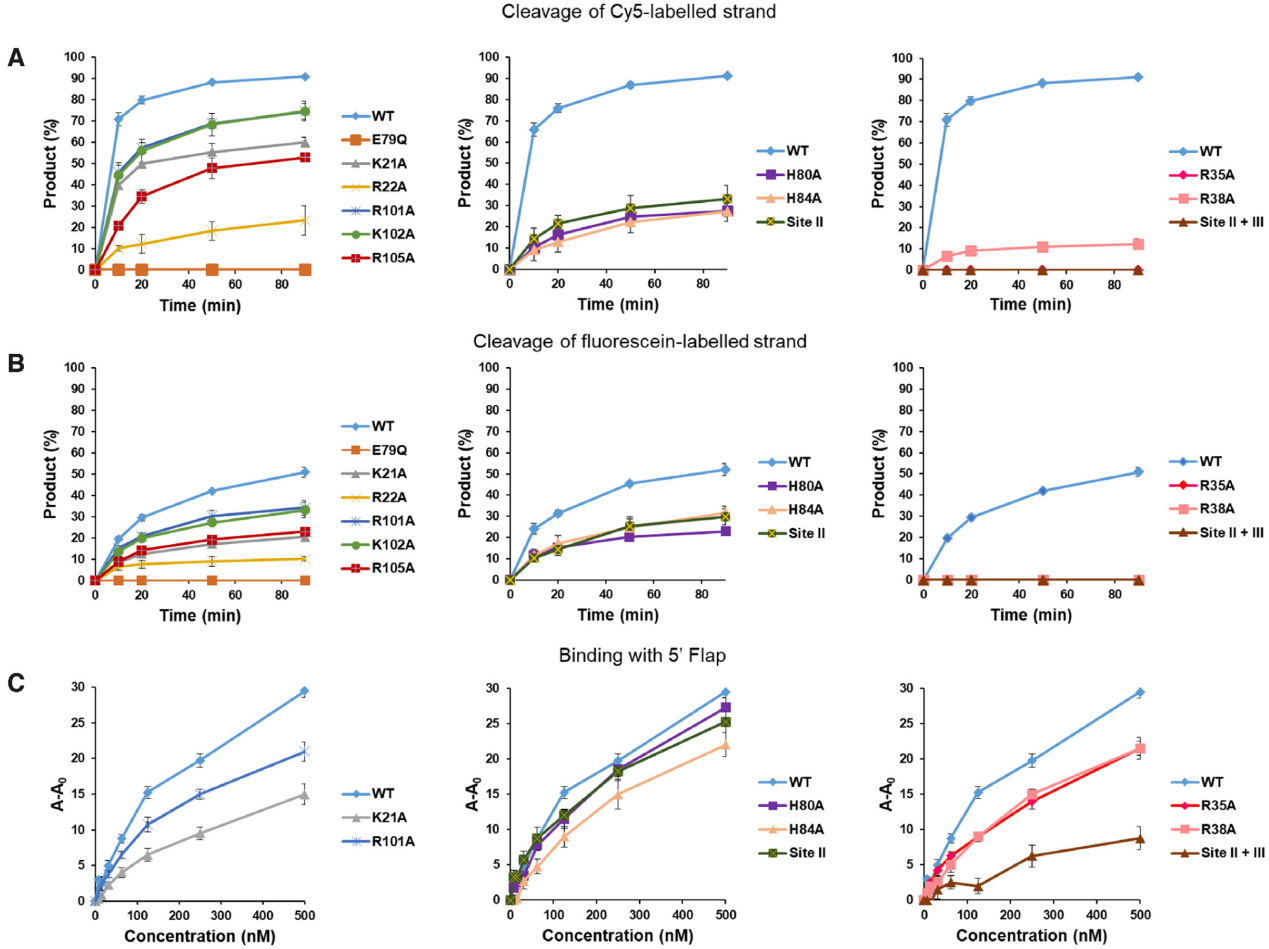
Activity and DNA binding by point mutations of *Cg*-Slx1 in complex with *Cg*-Slx4^CCD^. (**A**) Time course reactions of 5′-flap cleavage by *Cg*-Slx1–Slx4^CCD^ variants. Cleavage of the Cy5-labeled flap DNA strand was monitored. Cleavage products were resolved on denaturing gels and visualized by a fluorescence scanner. The amount of product was measured by gel densitometry and is reported as a percentage of total densitometry counts of all of the bands. (Left) Activity of site III mutants (*Cg*-Slx1^K21A^-Slx4^CCD^, *Cg*-Slx1^R22A^-Slx4^CCD^, *Cg*-Slx1^R101A^-Slx4^CCD^, *Cg*-Slx1^K102A^-Slx4^CCD^, *Cg*-Slx1^R105A^-Slx4^CCD^). (Middle) Activity of site II mutants (*Cg*-Slx1^H80A^-Slx4^CCD^, *Cg*-Slx1^H84A^-Slx4^CCD^, *Cg*-Slx1^H80A/H84A^-Slx4^CCD^). Mutant *Cg*-Slx1^H80A/H84A^-Slx4^CCD^ is referred to as the Site II mutant. (Right) Activity of site I mutants (*Cg*-Slx1^R35A^-Slx4^CCD^, *Cg*-Slx1^R38A^-Slx4^CCD^) and the *Cg*-Slx1^H80A/H84A/K21A/R22A/R101A/K102A^-Slx4^CCD^ variant with mutations in site II and site III (Site II + III). Wild type *Cg*-Slx1^WT^-Slx4^CCD^ and catalytically inactive Cg-Slx1^E79Q^-Slx4^CCD^were used as positive and negative controls, respectively. (**B**) As in (A) but cleavage of the fluorescein-labeled continuous strand was monitored. The representative gels that are related to the activity tests are shown in Supplementary Figure S5. All of the experiments were repeated three times. All variants were tested in each repetition and the results are split into separate panels for clarity. The plot for wild-type protein is repeated in each panel for easier comparisons. Error bars represent the standard deviation over three experiments. (**C**) Binding of 5′-flap DNA by selected site I, site II, and site III *Cg*-Slx1–Slx4 mutants. Binding was studied using fluorescence anisotropy. Cy5-labeled DNA strand was monitored. Descriptions of the various mutants are provided in Supplementary Table S3. Y-axis is labeled A-A_0_, where A is the measured anisotropy and A_0_ is the anisotropy of the DNA alone.

We then performed mutagenesis studies to assess the importance of various DNA-binding elements. These experiments were performed with the well-characterized *C. glabrata* protein (21). We first tested the importance of the new DNA-binding interface (site III). Residues that corresponded to the DNA-binding interface in *Cg-*Slx1 (Supplementary Figure S4) were individually substituted with alanine, resulting in K21A, R22A, R101A, K102A and R105A variants. Similar to wild type proteins, these Slx1 variants were co-purified with *Cg*-Slx4^CCD^. Wild type Slx1 and the E79Q variant were used as controls (Supplementary Table S3). E79 was predicted to coordinate the catalytic metal ion and E79Q mutant is catalytically inactive as shown previously (21). The catalytic activity of all of these variants was tested using the 5′ -flap substrate (Figure 5, Supplementary Figure S5). The results showed that each variant had lower activity. The R22A variant exhibited the most severe defect (20% of flap cleavage compared with 90% for wild type protein). Interestingly, cleavage of the flap strand was much more efficient (90% cleavage by wild type protein) than the cut of the continuous strand (50% cleavage). This is consistent with the function of Slx1 in flap removal. It is also consistent with our models of 5′-flap binding (Figure 4), which predicted that binding that is conducive to flap cleavage should involve site II and that cuts in the continuous strand would not involve site II. For the cut of the continuous strand, this strand continues toward site III and away from site II. For the cut in the flap strand, DNA that leaves the active site would continue toward site II, potentially forming additional interactions that enhance affinity and activity. To verify this, we tested the activity of site II mutants on both substrates. Consistent with our initial interpretation, mutations in site II affected cleavage of the continuous strand much less than the flap strand. For the site II variants a reduction from 50% to 30% product formation (after 90 min) was observed for continuous strand and a reduction from 90% to 30% for flap stand (Figure 5A and B; Supplementary Figure S5).

We next further explored the role of the three DNA-binding sites in Slx1. We used R35A and R38A variants with substitutions in site I, and another variant in which H80A and H84A substitutions were combined with the alanine replacement of four residues in site III: K21, R22, R101, K102 (when only site III was mutated, the protein was unstable; Supplementary Table S3). We obtained infrared spectra of these variants by Fourier-transform infrared spectroscopy (Supplementary Figure S6). The spectra were very similar to wild type protein, showing that the mutations did not alter protein structure. These variants were tested for activity on fluorescently labeled 5′-flap substrates (Figure 5A and B). We also measured the binding of Slx1–Slx4^CCD^ variants to 5′-flap DNA using fluorescence anisotropy (Figure 5C). The activity tests showed that any mutation in site I (R35A or R38A variant) resulted in the complete or nearly complete lack of activity (Figure 5A and B). This confirmed our previous findings (21) that site I is critical for enzymatic activity. In contrast, DNA binding was only mildly affected by the R35A and R38A mutations (Figure 5C). These results suggest that site II and site III can bind DNA independently of site I. This binding by sites II and III alone is not catalytic as the interaction of the DNA with site I is essential to place the scissile phosphate in the active site. Either individual or binary mutations in site II (H80A and H84A) led to a moderate defect in 5′-flap binding in agreement with small defect in continuous strand cleavage (Figure 5). The variant in which both site II and site III were eliminated did not have any enzymatic activity and showed weak binding of both substrates. Thus, site I was insufficient for stable DNA binding by *Cg*-Slx1–Slx4^CCD^ and most likely represented a low-affinity DNA-binding site (Figure 5C). Therefore, sites II and III were required to hold the substrate in place and assist binding in site I. The role of sites II and III is likely to prevent Slx1 from cleaving regular double-stranded DNA that would bind to site I alone. This can be further ensured by the safety latch mechanism that was described earlier, which potentially links DNA binding at site III with organization of the active site (Figure 2).

In summary, we found that Slx1 contained three DNA-binding interfaces. Site I was key for catalytic orientation of the DNA. Site III was a general DNA-binding interface, whereas site II played a more specialized role in enhancing certain activities (e.g. cleaving the flap strand in 5′-flap substrates).

## DISCUSSION

Structure-selective endonucleases are thought to require tight regulation in cells because the unrestrained DNA cleavage of branched DNA structures would destabilize the genome. This regulation can be achieved by protein-protein interactions, post-translational modifications, and cell cycle-dependent activation, among others (30,31). Additionally, nearly all SSEs have mechanisms for the proper and specific cleavage of their substrates at desired sites (for review, see (32)). This stringency is enforced by the presence of specific structural features in the nuclease itself, which allows the binding of only specific DNA substrates. For example, the homodimeric bacterial HJ resolvase RuvC specifically binds all four arms of the HJ to position two phosphate groups that are symmetrically located 1 nt from the branch point in the two active sites. RuvC also cleaves DNA at a particular cognate sequence (33,34). FEN1 bends the DNA, recognizes a 1nt 3′ -flap, threads the 5′ -flap under a special structure termed the helical arch, and frays the ends of the DNA duplex for specific cleavage (35,36). MUS81-EME1 has a 5′-phosphate-binding pocket to position nicked HJs for cleavage in the strand that is opposite to the nicked strand (15).

Slx1 is quite different, in which it cleaves many diverse types of branched DNA structures and at different positions near the branch point (14,20,21). In agreement with this, our structures of *Cg*-Slx1–Slx4^CCD3^ and *Tt*-Slx1–Slx4^CCD3^ did not reveal any features that could be related to the specific binding of particular DNA structures. We could only identify patches of positively charged residues binding DNA substrate in a specific orientation around the catalytic site to facilitate catalysis. Therefore, an intriguing issue is the way in which Slx1 interacts with various DNA substrates.

Our structural and biochemical work identified three positively charged patches on the surface of Slx1, designated sites I, II and III ((21) and present study). Site I is postulated to play a crucial role in binding and orienting the substrate for catalysis. However, site I is insufficient for DNA cleavage and requires assistance from site II and site III. This is an important feature of the mechanism of Slx1 because it can explain why the enzyme is unable to cleave linear duplex DNA, which would only bind site I. A complex interplay of interactions between DNA and sites II and III allows Slx1 to bind a wide range of branched DNA substrates, thus making Slx1 a very promiscuous enzyme. The orientation of site III relative to site I is such that DNA bending is essential for effective catalysis. This bending helps localize the branch point, which itself represents a malleable discontinuity in the DNA substrate. This discontinuity is essential for catalysis and thus provides a safeguard to protect the genome from otherwise highly active nucleases. Interestingly, the GIY-YIG-containing C-terminal domain of MSH1 from *Arabidopsis thaliana* has been shown to bind branched DNA structures that represent homologous recombination intermediates. No nuclease activity has been observed for the GIY-YIG domain of *Arabidopsis* MSH1 (37), but these results indicate that other GIY-YIG domains can bend the substrate. The structures that are presented herein illuminate another safeguard against the indiscriminate cleavage of DNA by Slx1. Slx1 follows a disorder-to-order transition mechanism for organizing the catalytic site for processing joint DNA molecules. In fact, Slx1 is not the only structure-selective endonuclease that exhibits this mechanism - a substrate-induced disordered-to-ordered transition has also been observed in the case of FEN1 (36).

Slx1 is active only in the presence of Slx4. We previously reported the role of Slx4 in the activation of *Cg*-Slx1, which otherwise exists as an inactive homodimer (21). Our structures and models reveal that Slx4 is present away from the trajectories of modeled DNA and indicate that it is not involved in DNA binding. However, the role of Slx4 in binding DNA substrates needs to be explored further, especially because mammalian SLX4 has been shown to be involved in the coordination of SLX1 and MUS81-EME1 (20,38).

Slx1 is a very promiscuous endonuclease and needs to be tightly regulated. Our present and previous (21) work identified three mechanisms to achieve such regulation: (i) the interaction with Slx4 is essential for the activation of Slx1, (ii) the branch point of DNA is specifically found by DNA bending, and (iii) the reorganization of Slx1 upon interactions with DNA substrate potentially leads to active site formation.

## DATA AVAILABILITY

The structures of *Tt*-Slx1^E79Q^-Slx4^CCD3^ alone and in complex with the DNA were deposited in the PDB under the accession codes 6SEH and 6SEI, respectively.

## Supplementary Material

gkz842_Supplemental_File

## Notes

Present address: Vineet Gaur, National Institute of Plant Genome Research, Aruna Asaf Ali Marg, New Delhi 110067, India.
