# Supplementary material for: Recognition and processing of branched DNA substrates by Slx1–Slx4 nuclease
Source: Nucleic Acids Res. 2019 Oct 4;47(22):11681–90. doi: 10.1093/nar/gkz842 (PMC6902002; doi:10.1093/nar/gkz842)
Supplement: gkz842_Supplemental_File [file gkz842_supplemental_file.pdf]

SUPPLEMENTARY INFORMATION

Recognition and processing of branched DNA substrates by Slx1-Slx4 nuclease

Vineet Gaur,[1,&] Weronika Ziajko,[1] Shivlee Nirwal,[1] Aleksandra Szlachcic,[1]

Marta Gapińska,[1] and Marcin Nowotny[1,*]

[1] Laboratory of Protein Structure, International Institute of Molecular and Cell Biology, 4 Trojdena St., 02-109 Warsaw, Poland

[&] Current Address: National Institute of Plant Genome Research, Aruna Asaf Ali Marg, New Delhi 110067, India

* To whom correspondence should be addressed. e-mail: mnowotny@iimcb.gov.pl

SUPPLEMENTARY FIGURES

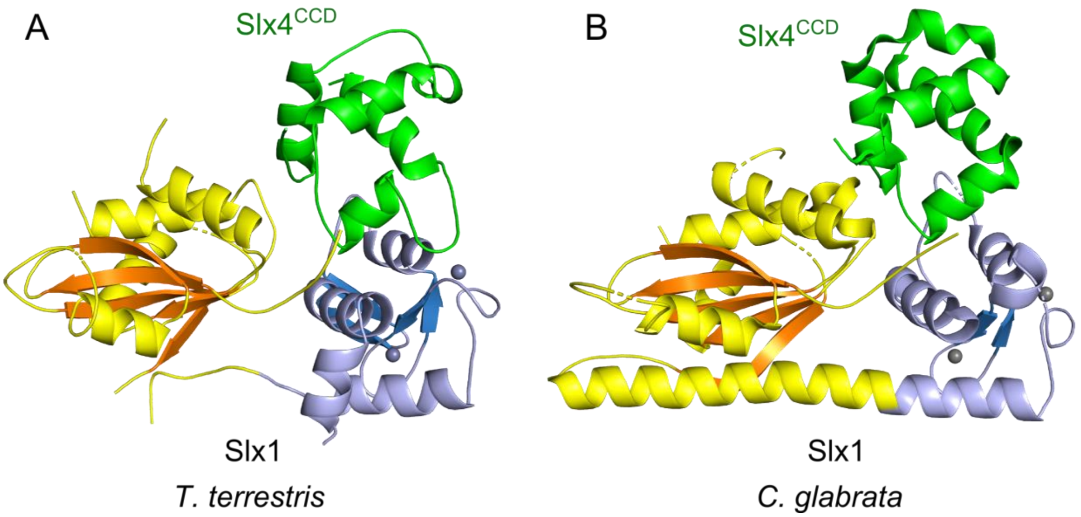

Supplementary Figure S1. Overall structure of apo Slx1-Slx4^{CCD3}. (A) structure of *Tt*-Slx1-Slx4^{CCD3} (present study). Slx1 nuclease domain is shown in yellow with β-strands in orange. RING domain is shown in blue. Slx4 is shown in green. Zinc ions are shown as gray spheres. (B) *C. glabrata* Slx1-Slx4^{CCD3} (PDB ID: 4XLG).

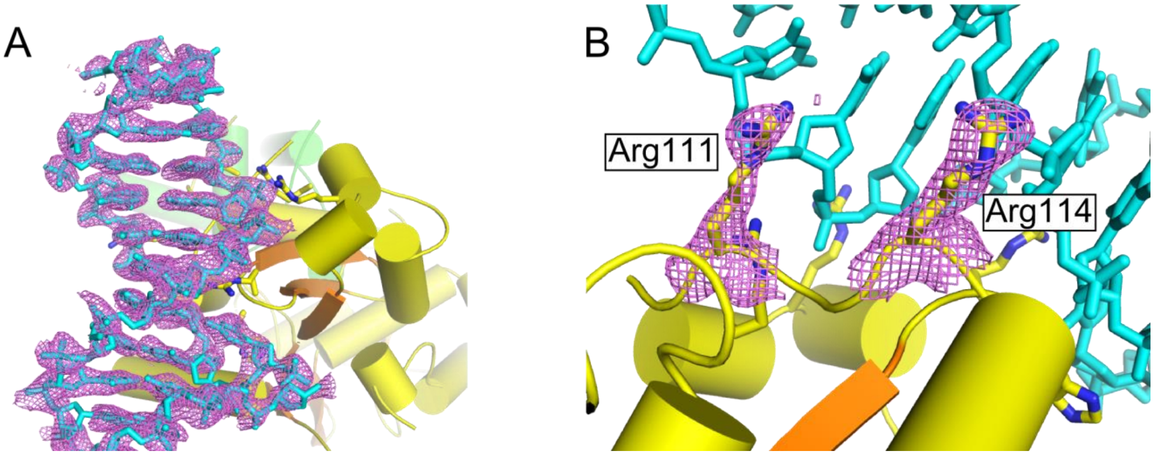

Supplementary Figure S2. Simulated annealing composite-omit maps for *Tt*-Slx1-Slx4^{CCD3}–DNA structure. 2Fo-Fc map contoured at 1.1 σ is shown for the DNA model (**A**) and selected DNA-binding residues (**B**).

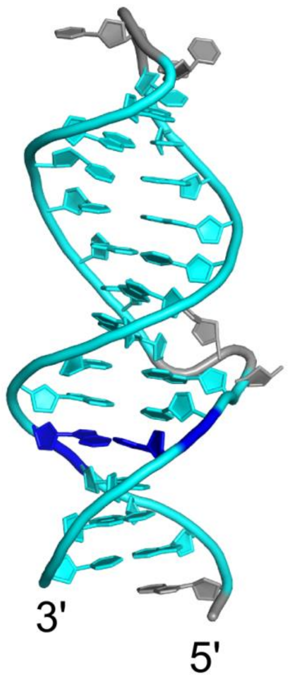


```
 T
T T
C-G
C-G
A-T
G-C
T-A
A-T
A-T
| C
| G
C-G
G-C
G A
C-G
T-A
A-T
A |
C 3'
|
5'
```

Supplementary Figure S3. Structure of the DNA. (Left) Structure of the DNA from the *Tt*-Slx1-Slx4^{CCD3}–DNA structure. The DNA is shown in cyan for canonical base pairs, in blue for single G-A mismatch and in gray for unpaired bases (Right) Schematic of base pairing in this DNA.

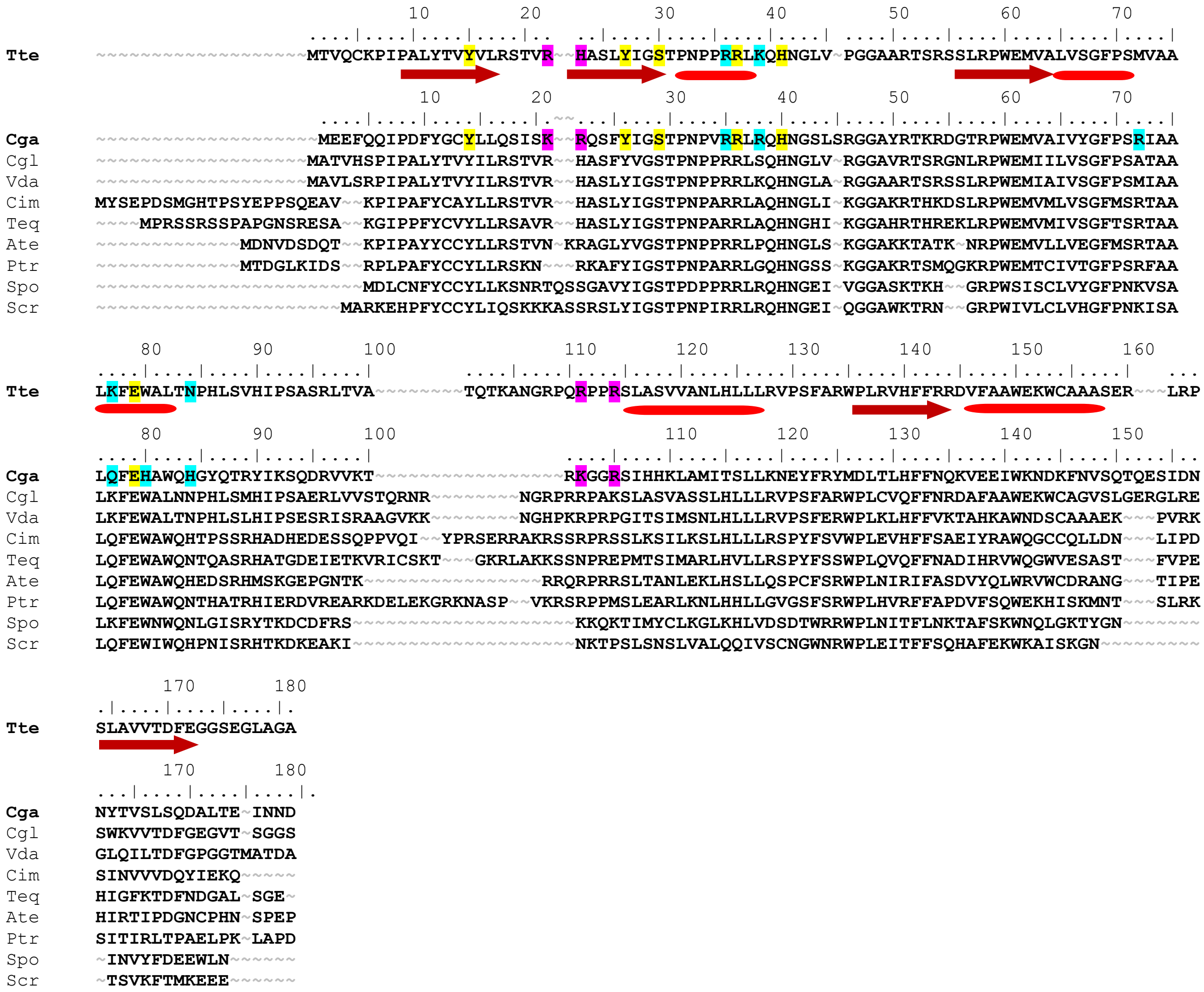

Supplementary Figure S4. Multiple sequence alignment of nuclease domains of fungal Slx1 proteins. Secondary structure elements are shown for *Tte*-Slx1 (arrows for β-stands and cylinders for α-helices). Residues that are involved in DNA binding at the new interface are highlighted in purple. Active-site residues are shown in yellow. Predicted site I and II residues are shown in cyan. Tte, *Thelavia terrestris*; Cga, *Candida glabrata*; Pgt, *Puccinia graminis tritici*; Cgl, *Chaetomium globosum*; Vda, *Verticillium dahlia*; Cim, *Coccidioides immitis*; Teq, *Trichophyton equinum*; Ate, *Aspergillus terreus*; Ptr, *Pyrenophora triticirepentis*; Spo, *Schizosaccharomyces pombe*; Scr, *Schizosaccharomyces cryophilus*.

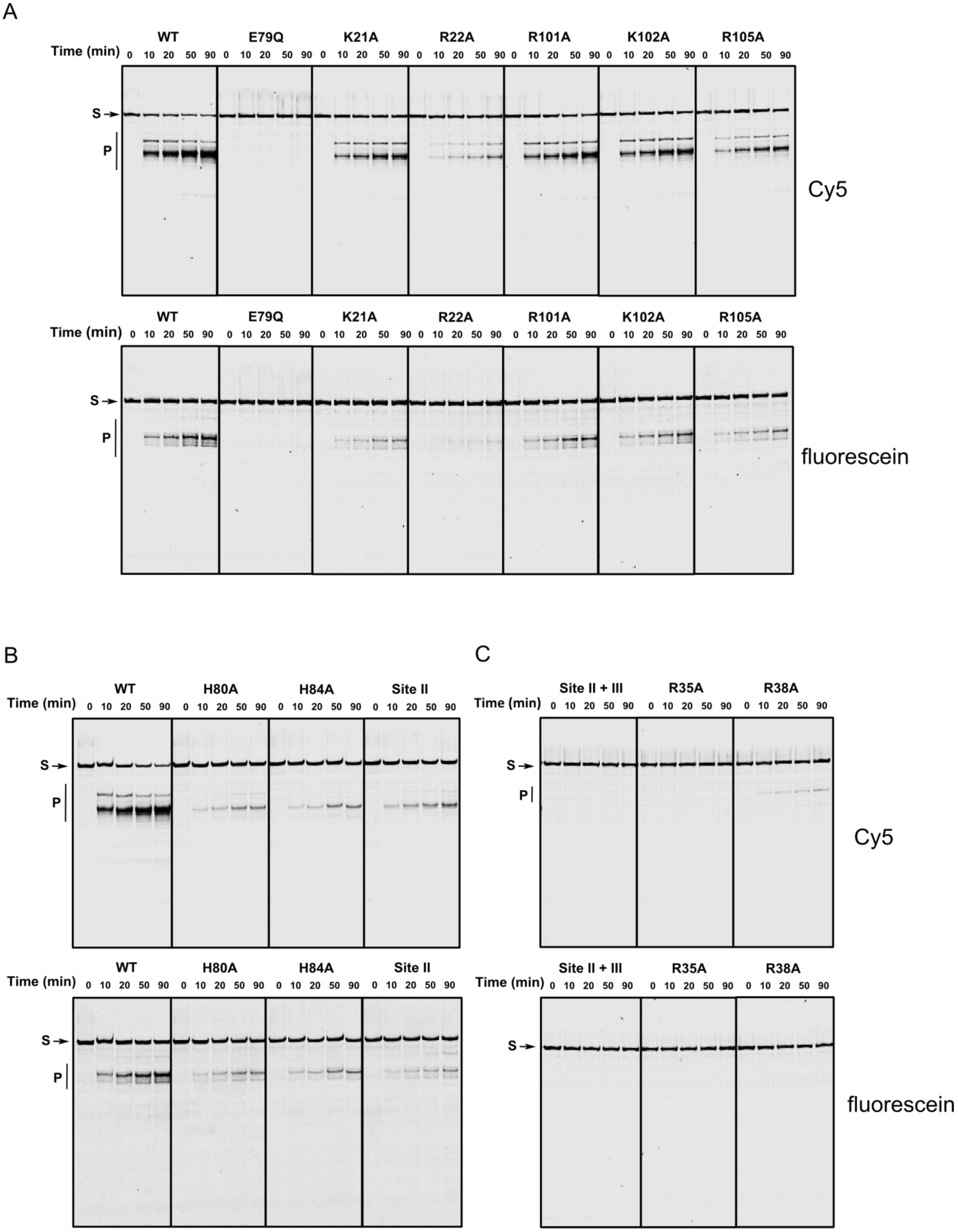
A
WT
E79Q
K21A
R22A
R101A
K102A
R105A
Time (min)
0 10 20 50 90
S
P
Cy5
fluorescein
B
H80A
H84A
Site II
C
Site II + III
R35A
R38A

Supplementary Figure S5. Representative gels that were used to quantify the activity of various mutants of *Cg*-Slx1-Slx4^{CCD} on 5'-flap substrate. (**A**) Activity test of site III mutants. (**B**) Activity test of site II mutants. (**C**) Activity tests of site I mutants together with a mutant in which site II and site III were mutated. Upper gels in each panel present the activity of the Cy5-labeled strand. Lower gels in each panel present the activity of the fluorescein-labeled strand. Bands corresponding the substrate (S) and products of the reaction (P) are labeled on the right side of the panels.

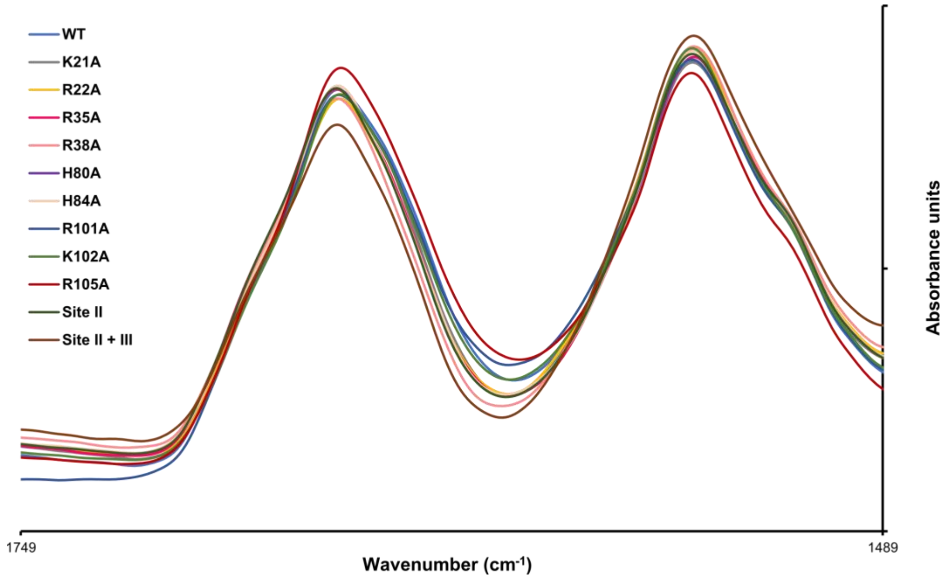

Supplementary Figure S6. Secondary structure content and structural integrity of *Cg*-Slx1-Slx4^{CCD} variants. Fourier-Transform Infrared (FT-IR) spectrum of *Cg*-Slx1-Slx4^{CCD} and various mutants (Supplementary Table S3)**.**

Supplementary Table S1. Sequences of oligonucleotides (written 5′ to 3′).

**Oligonucleotides used to generate substrates for activity test and binding studies.**	
X0-1	ACGCTGCCGAATTCTACCAGTGCCTTGCTAGGACATCTTTGCCCACCTGCAGGTTCACCC
X0-2	GGGTGAACCTGCAGGTGGGCAAAGATGTCCATCTGTTGTAATCGTCAAGCTTTATGCCGT
X0-3	ACGGCATAAAGCTTGACGATTACAACAGATCATGGAGCTGTCTAGAGGATCCGACTATCG
X0-4	CGATAGTCGGATCCTCTAGACAGCTCCATGTAGCAAGGCACTGGTAGAATTCGGCAGCGT
X0-1[f]	(Fluorescein)ACGCTGCCGAATTCTACCAGTGCCTTGCTAGGACATCTTTGCCCACCTGCAGGTTCACCC
X0-4[c]	CGATAGTCGGATCCTCTAGACAGCTCCATGTAGCAAGGCACTGGTAGAATTCGGCAGCGT (Cy5)
X0-1[fc]	(Fluorescein)ACGCTGCCGAATTCTACCAGTGCCTTGCTAGGACATCTTTGCCCACCTGCAGGTTCACCC (Cy5)
X0-2.30	GGGTGAACCTGCAGGTGGGCAAAGATGTCC
X0-3.30	CATGGAGCTGTCTAGAGGATCCGACTATCG
X0-4.30	TAGCAAGGCACTGGTAGAATTCGGCAGCGT
**Oligonucleotides used for mapping cleavage sites**	
X0-1.28[f]	(Fluorescein)ACGCTGCCGAATTCTACCAGTGCCTTGC
X0-1.29[f]	(Fluorescein)ACGCTGCCGAATTCTACCAGTGCCTTGCT
X0-1.31[f]	(Fluorescein)ACGCTGCCGAATTCTACCAGTGCCTTGCTAG
X0-1.32[f]	(Fluorescein)ACGCTGCCGAATTCTACCAGTGCCTTGCTAGG
X0-4.28[c]	GCAAGGCACTGGTAGAATTCGGCAGCGT(Cy5)
X0-4.29[c]	AGCAAGGCACTGGTAGAATTCGGCAGCGT(Cy5)
**Oligonucleotides used for crystallization**	
114a	CAATCGGCAATGACCTTTGGTCATTCAGCAGAT
114b	ATCTGCTGAATCTGGTTTCCAGATTGCCGATTG

Supplementary Table S2. Oligonucleotides that were used to generate DNA substrates.

Substrates	Unlabeled	Labeled
**Substrates used in crystallization**		
Holliday junction	114a, 114b	
Splayed-arm	114a	
**Substrates used in activity test/fluorescence anisotropy**		
Holliday junction	X0-1, X0-2, X0-3, X0-4	Fluorescent: X0-1[f], X0-2, X0-3, X0-4[c]
Replication fork	X0-1, X0-2.30, X0-3.30, X0-4	Fluorescent: X0-1[f], X0-2.30, X0-3.30, X0-4[c]
5′-flap	X0-1, X0-2.30, X0-4	Fluorescent: X0-1[f], X0-2.30, X0-4[c]
3′-flap	X0-1, X0-3.30, X0-4	Fluorescent: X0-1[f], X0-3.30, X0-4[c]
Splayed-arm	X0-1, X0-4	Fluorescent: X0-1[f], X0-4[c]
Nicked DNA	X0-1, X0-2.30, X0-4.30	Fluorescent: X0-1[fc], X0-2.30, X0-4.30

[f] Fluorescein-labeled oligonucleotides. [c] Cy5-labeled oligonucleotides. [fc] Oligonucleotide labeled with Fluorescein at 5′ end and Cy5 at 3′ end.

Supplementary Table S3. ***Cg*****-Slx1-Slx4^{CCD} mutants that were used in the study.**

	**Mutant name**	
	**Mutations in Site I**	
1	R35A	*Cg*-Slx1^{R35A}-Slx4^{CCD}
2	R38A	*Cg*-Slx1^{R38A}-Slx4^{CCD}
	**Mutations in Site II**	
1	H80A	*Cg*-Slx1^{H80A}-Slx4^{CCD}
2	H84A	*Cg*-Slx1^{H84A}-Slx4^{CCD}
3	Site II	*Cg*-Slx1$^{H80A/H84A}$-Slx4^{CCD}
	**Mutations in Site III**	
1	K21A	*Cg*-Slx1^{K21A}-Slx4^{CCD}
2	R22A	*Cg*-Slx1^{R22A}-Slx4^{CCD}
3	R101A	*Cg*-Slx1^{R101A}-Slx4^{CCD}
4	K102A	*Cg*-Slx1^{K102A}-Slx4^{CCD}
5	R105A	*Cg*-Slx1^{R105A}-Slx4^{CCD}
	**Mutations in Site II and Site III**	
1	Site I + II	*Cg*-Slx1$^{H80A/H84A/K21A/R22A/R101A/K102A}$-Slx4^{CCD}
	**Others**	
1	E79Q	*Cg*-Slx1^{E79Q}-Slx4^{CCD}
2	Wild type	*Cg*-Slx1-Slx4^{CCD}
